# High-fat diets containing different types of fatty acids modulate gut-brain axis in obese mice

**DOI:** 10.1186/s12986-022-00675-3

**Published:** 2022-06-23

**Authors:** Yinan Hua, Jingyi Shen, Rong Fan, Rong Xiao, Weiwei Ma

**Affiliations:** Beijing Key Laboratory of Environmental Toxicology, School of Public Health, Capital Medical University, No.10 Xitoutiao, You An Men Wai, Beijing, 100069 People’s Republic of China

**Keywords:** Fatty acid, High-fat diet, Intestinal barrier, Gut-brain axis, Obesity

## Abstract

**Background:**

Excessive consumption of high-fat diets is associated with disordered metabolic responses, which may lead to chronic diseases. High-fat diets containing different types of fatty acids lead to distinct alterations in metabolic responses of gut-brain axis.

**Methods:**

In our study, normal male C57BL/6J mice were fed to multiple high fatty acid diets (long-chain and medium-chain saturated fatty acid, LCSFA and MCSFA group; n-3 and n-6 polyunsaturated fatty acid, n-3 and n-6 PUFA group; monounsaturated fatty acid, MUFA group; trans fatty acid, TFA group) and a basic diet (control, CON group) for 19 weeks. To investigate the effects of high-fat diets on metabolic responses of gut-brain axis in obese mice, blood lipids were detected by fast gas chromatography, and related proteins in brain and intestine were detected using Western blotting, ELISA, and immunochemistry analysis.

**Results:**

All high-fat diets regardless of their fatty acid composition induced obesity, lipid disorders, intestinal barrier dysfunction, and changes in gut-brain axis related factors except basal diet in mice. For example, the protein expression of zonula occludens-1 (ZO-1) in ileum in the n-3 PUFA group was higher than that in the MCSFA group (*P* < 0.05). The expressions of insulin in hippocampus and leptin in ileum in the MCSFA group significantly increased, compared with other groups (all *Ps* < 0.05).

**Conclusion:**

The high MCSFA diet had the most effect on metabolic disorders in gut-brain axis, but the high n-3 PUFA diet had the least effect on changes in metabolism.

## Introduction

Accumulating evidence have suggested that different dietary components may modulate gene expression and metabolic responses, which may in turn lead to increased risk of chronic diseases including obesity, lipid disorders, insulin resistance, cognitive dysfunction, etc. [1]. Numerous animal studies demonstrated that high-fat diet induced obesity, even influenced cognitive function [2, 3]. Obesity has been involved in hippocampal atrophy and cognitive dysfunction in human [4, 5]. Epidemiological studies showed that increased consumption of polyunsaturated and monounsaturated fats was irrelevant to weight change, but increased consumption of saturated and trans fats was positively correlated to weight gain [6].

In addition to obesity, high-fat diet may also influence the structure and function of intestinal epithelial barrier [7] and central nervous system. Yang et al*.* showed that high-fat diet decreased the mRNA expression of gut tight junction protein occludin, claudin-3, zonula occludens-1 (ZO-1) [8], which formed a selectively permeable seal between adjacent cells [9] to protect intestinal mucosal barrier. Destroyed permeability and integrity of intestinal barrier may lead to release of bacteria, toxins, and other molecules into blood [10], which may induce gut–brain axis imbalance, cognitive function and behavior of obese individuals [11].

Furthermore, some studies indicated that different fatty acids induced different alterations in gut–brain axis, even on the cognitive function in obese subjects. Wu et al*.* demonstrated that high saturated fat diet reduced the expression of brain-derived neurotrophic factor (BDNF) in SD rats [12]. Related researches indicated that BDNF expression increased in n-3 polyunsaturated fatty acid (PUFA) adequate rats, which might be due to upregulating effects of n-3 PUFA on BDNF and its receptor [13]. Other studies indicated that saturated fatty acid increases plasma leptin in humans [14], which was associated with the occurrence of Alzheimer’s disease (AD) [15]. Kristine et al*.* [16] showed that PUFA-rich diets reduced the ghrelin release to suppress postprandial hunger. However, there is a paucity of cross-sectional comparisons of the effects among different dietary fatty acids on gut–brain axis.

In the present study, we examined the influence of different dietary fatty acids on gut–brain axis in obese C57BL/6J mice. The aim of our study is to compare the effects of various high dietary fat acid diets exhibit on metabolic responses across gut–brain axis in obese mice.

## Materials and methods

### Animals and diets

C57BL/6J male mice (aged 4 week-old), purchased from Beijing Vital River Laboratory Animal Technology Co., Ltd. (Beijing, China, SCXK—(Jing) 2016-0006), were housed at room temperature (22 ± 1 °C) under a 12-h light–dark cycle at the SPF Laboratory Animal Center. After 4 weeks of acclimation, mice were randomly assigned into seven weight-matched groups (n = 10 per group; group-housed 2 per cage): control group on chow diet (CON group, 10% of energy derived from fat research Diets D12450H) and other groups on high saturated fatty acids (long-chain: lard oil; medium-chain: coconut oil), polyunsaturated fatty acids (n-3: flaxseed oil; n-6: soybean oil), monounsaturated fatty acid (olive oil), and trans fatty acid (hydrogenated soybean oil) diets (LCFSA, MCSFA, n-3 PUFA, n-6 PUFA, MUFA, and TFA group, respectively, 45% of energy derived from fat research Diets D12451) for 19 consecutive weeks. Mice were weighed weekly and observed daily.

At the end of study, overnight fasted mice were anaesthetized with 5% chloral hydrate, and the blood was collected with EDTA anticoagulation tube. The lipid profiles in plasma were measured using fast gas chromatography analysis on a Shimadzu GC-2010 Gas Chromatograph (Shimadzu, Japan). The collected fat tissues (perirenal fat, peri-testicular fat, and omental fat) were weighted. The brain, ileum, and colon tissues were collected for subsequent analysis. All animal procedures were approved by Animal Care and Ethics Committee of Capital Medicine University (Ethics Review No: AEEI-2018-061).

### Western blotting

The brain, colon, and ileum tissues were lysed in Radio Immunoprecipitation Assay (RIPA) lysis buffer (Roby, China). BCA total protein assay kit was used to determine the concentration of protein (Nanjing Jiancheng, China). Equal amounts of protein extracts (50ug) were separated by 12% SDS–polyacrylamide gel, and transferred onto polyvinylidene difluoride membrane (Millipore, USA). The membrane was blocked with 5% nonfat milk for 1 h at room temperature, and then incubated with primary antibodies for β-actin (Cell Signaling Technology, USA), claudin-1, claudin-5, occludin, and ZO-1 (Abcam, USA) overnight at 4 ℃. Subsequently, at room temperature, the membrane was incubated with horseradish peroxidase conjugated secondary antibodies (Cell Signaling Technology, USA) for 1 h. The bands were detected with an enhanced chemiluminesence western blotting kit (Keygen Biotech, China) and exposed to Fusion Fx film (Vilber Lourmat, France). The results were quantified as the ratio of relative gray value of target protein to the internal control, β-actin.

### ELISA and immunohistochemistry staining

The concentrations of BDNF and serotonin (5-HT) levels in the brain, colon and ileum were measured by ELISA kits (Mlbio, China) according to the manufacturer’s protocol. The level of ghrelin, insulin, and leptin in the brain, and the levels of ghrelin and leptin in the ileum and colon issues were detected by immunohistochemistry. Specimens were dehydrated, cleared, and paraffin-embedded. The sections were conventionally dewaxed, washed, and incubated in 3% H_2_O_2_ to quench endogenous peroxidase activity. Next, the sections were incubated with specific primary antibodies (ghrelin, insulin, and leptin, Servicebio, China) and second antibodies (Servicebio, China). The immunoreaction was visualized by DAB reagents and nucleus was lightly counterstained with hematoxylin. Then the sections were dehydrated and mounted. We measured the sections using Image-Pro Plus 6.0 image analysis system and examined the average optical density values.

### Statistical analysis

Data were presented as mean ± standard deviation (SD) using SPSS 23.0 software. The differences among all groups were analyzed by one-way ANOVA followed using Fisher’s protected least significant difference (LSD) or Dunnett’s T3 and visualized with GraphPad Prism version 6.0. The two-sided significance level of the test was 0.05.

## Results

### Changes in the body weight and body fat

**Fig. 1:**
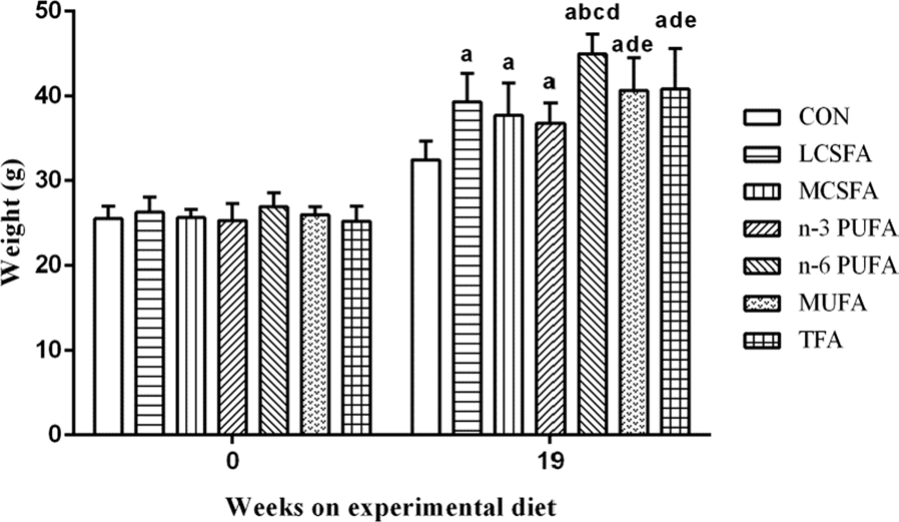
Body weight of mice at the 0 and 19th week of intervention. n = 10, data expressed as the mean ± SD. a *P* < 0.05, compared to CON group; b: *P* < 0.05, compared to LCSFA group; c: *P* < 0.05, compared to MCSFA group; d: *P* < 0.05, compared to n-3 PUFA group; e: *P* < 0.05, compared to n-6 PUFA group

Figure 1 showed that the body weight of mice at the beginning and the end of dietary treatment. No significant differences were shown at the beginning of treatment in mice (all *Ps* > 0.05). At the 19th week, the body weights of mice in high-fat groups were all higher than that in the CON group (all *Ps* < 0.05). The weight of mice in the n-6 PUFA was significantly higher than that in the LCSFA, MCSFA, n-3 PUFA, MUFA, and TFA groups (all *Ps* < 0.05). Mice in the n-3 PUFA group had lower body weight than that in the MUFA group (*P* < 0.05). The body weight of mice in the TFA group was higher than that in the n-3 PUFA group (*P* < 0.05).

**Fig. 2:**
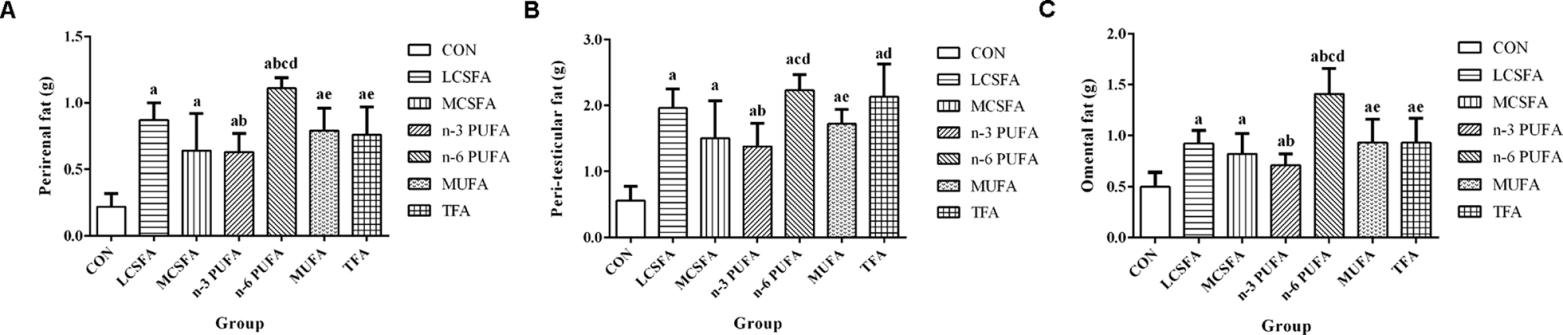
Effect of diets on the body fat mass in obese mice. **A** Comparison of the levels of perirenal fat among the seven groups. **B** Comparison of the levels of peri-testicular fat among the seven groups. **C** Comparison of the levels of omental fat among the seven groups. n = 10, data expressed as the mean ± SD. a: *P* < 0.05, compared to CON group; b: *P* < 0.05, compared to LCSFA group; c: *P* < 0.05, compared to MCSFA group; d: *P* < 0.05, compared to n-3 PUFA group; e: *P* < 0.05, compared to n-6 PUFA group

As shown in Fig. 2, compared with the CON group, mice had higher contents of perirenal fat, peri-testicular fat, and omental fat in high-fat groups (all *Ps* < 0.05). The perirenal fat in the n-6 PUFA group was higher than those in other groups (all *Ps* < 0.05). The perirenal fat in the LCSFA group was higher than that in the n-3 PUFA groups (*P* < 0.05). The peri-testicular fat in the LCSFA, n-6 PUFA, and TFA groups was higher than that in the n-3 PUFA group (*P* < 0.05). Compared to that in the n-6 PUFA group, the MCSFA and MUFA groups had lower peri-testicular fat contents (all *Ps* < 0.05). In addition, compared with that in the n-6 PUFA group, other groups had lower omental fat contents (all *Ps* < 0.05). The omental fat in the n-3 PUFA was lower than that in the LCSFA group (*P* < 0.05).

In conclusion, the n-6 PUFA-rich diet had the greatest effect on the increase in the body weight and body fat of mice, while the n-3 PUFA-rich diet had the least effect.

### Changes in the lipid profiles of plasma

As shown in Table 1, the lipid profiles in plasma of mice were assayed. Compared with those in the CON group, the concentrations of C18:2 n-6c, C20:4 n-6, total n-6 PUFA, and total PUFA increased in the LCSFA group. In contract, the concentrations of C22:0, C16:1, C18:1 n-9c, and total MUFA decreased in the LCSFA group; the concentrations of C12:0, C14:0, C16:1, C18:1 n-9c, total MUFA, and C18:2 n-6c increased in the MCSFA group (all *Ps* < 0.05). The concentrations of C18:2 n-6c, C18:3 n-3, C20:5 n-3, C22:6 n-3, total n-3 PUFA, and total PUFA in the n-3 PUFA group were higher than those in the CON group. Conversely, C16:0, C22:0, total SFA, C16:1, C18:1 n-9c, total MUFA, C20:3 n-6, C20:4 n-6, and n-6/n-3 PUFA of the n-3 PUFA group were lower (all *Ps* < 0.05). Compared with those in the CON group, the contents of C18:2 n-6c, total n-6 PUFA, C22:6 n-3, total n-3 PUFA, and total PUFA increased in the n-6 PUFA group. Conversely, the contents of C22:0, C16:1, C18:1 n-9c, and total MUFA decreased in the n-6 PUFA group. Compared with the CON group, the concentrations of C18:1 n-9c, total MUFA, C18:2 n-6c, C20:4 n-6, total n-6 PUFA, C22:6 n-3, total n-3 PUFA, and total PUFA increased, but C22:0, and C16:1 decreased in the MUFA group. Moreover, the contents of C18:2 n-6c, C20:4 n-6, total n-6 PUFA, C22:6 n-3, total n-3 PUFA, total PUFA, and C18:1 n-9t (TFA) in the TFA group were higher those that in the CON group, while C22:0 and C16:1 were lower than those in the CON group (all *Ps* < 0.05). In addition, according to the data in Table 1, we found that the contents of total SFA and the n-6/n-3 PUFA ratio were the highest, as well as the content of total n-3 PUFA was the lowest of the plasma in the MCSFA group. But the mice of the n-3 PUFA group presented the opposite situation.

**Table 1:** Effect of diets on the lipid profiles of plasma

Fatty acid (mg/ml)	CON	LCSFA	MCSFA	n-3 PUFA	n-6 PUFA	MUFA	TFA	*F*	*P*
C8:0	0.000 ± 0.000	0.008 ± 0.015	0.000 ± 0.000	0.000 ± 0.000	0.000 ± 0.000	0.008 ± 0.021	0.000 ± 0.000	1.162	0.342
C12:0	0.000 ± 0.000	0.008 ± 0.022	0.031 ± 0.008^a^	0.000 ± 0.000^c^	0.002 ± 0.005^c^	0.000 ± 0.000^c^	0.003 ± 0.007^c^	11.883	** < 0.001**
C13:0	0.000 ± 0.000	0.000 ± 0.000	0.000 ± 0.000	0.000 ± 0.000	0.000 ± 0.000	0.000 ± 0.000	0.007 ± 0.018	1.000	0.436
C14:0	0.005 ± 0.004	0.002 ± 0.003	0.043 ± 0.008^ab^	0.002 ± 0.002^c^	0.005 ± 0.009^c^	0.003 ± 0.003^c^	0.003 ± 0.003^c^	63.585	** < 0.001**
C15:0	0.000 ± 0.000	0.000 ± 0.000	0.001 ± 0.001	0.000 ± 0.000	0.000 ± 0.000	0.000 ± 0.000	0.000 ± 0.000	1.750	0.129
C16:0	0.692 ± 0.082	0.668 ± 0.127	0.751 ± 0.063	0.507 ± 0.089^abc^	0.613 ± 0.087^c^	0.691 ± 0.126^d^	0.648 ± 0.155^d^	4.060	**0.002**
C17:0	0.001 ± 0.001	0.003 ± 0.003	0.000 ± 0.000	0.001 ± 0.002	0.001 ± 0.003	0.001 ± 0.002	0.001 ± 0.002	1.232	0.306
C18:0	0.408 ± 0.056	0.433 ± 0.123	0.406 ± 0.021	0.369 ± 0.061	0.413 ± 0.084	0.406 ± 0.094	0.381 ± 0.093	0.522	0.789
C20:0	0.000 ± 0.000	0.000 ± 0.000	0.001 ± 0.001	0.000 ± 0.000	0.000 ± 0.000	0.000 ± 0.000	0.000 ± 0.000	1.000	0.436
C22:0	0.101 ± 0.032	0.000 ± 0.000^a^	0.120 ± 0.041^b^	0.000 ± 0.000^ac^	0.000 ± 0.000^ac^	0.028 ± 0.014^abcde^	0.002 ± 0.006^acf^	51.476	** < 0.001**
Total SFA	1.207 ± 0.123	1.121 ± 0.245	1.352 ± 0.107^b^	0.879 ± 0.144^abc^	1.033 ± 0.168^c^	1.136 ± 0.227^ cd^	1.044 ± 0.243^c^	5.016	** < 0.001**
C14:1	0.000 ± 0.000	0.003 ± 0.007	0.001 ± 0.002	0.000 ± 0.000	0.000 ± 0.000	0.000 ± 0.000	0.000 ± 0.000	1.021	0.423
C15:1	0.000 ± 0.000	0.001 ± 0.003	0.000 ± 0.000	0.000 ± 0.000	0.000 ± 0.000	0.000 ± 0.000	0.000 ± 0.000	1.000	0.436
C16:1	0.090 ± 0.019	0.038 ± 0.012^a^	0.183 ± 0.037^ab^	0.020 ± 0.008^ac^	0.021 ± 0.006^ac^	0.039 ± 0.008^acde^	0.052 ± 0.013^acde^	85.237	** < 0.001**
C17:1	0.000 ± 0.000	0.001 ± 0.003	0.003 ± 0.006	0.004 ± 0.006	0.000 ± 0.000	0.003 ± 0.006	0.000 ± 0.000	1.512	0.194
C18:1 n-9c	0.389 ± 0.050	0.264 ± 0.066^a^	0.467 ± 0.090^ab^	0.155 ± 0.029^abc^	0.163 ± 0.037^abc^	0.538 ± 0.072^abcde^	0.360 ± 0.051^bcdef^	47.979	** < 0.001**
C20:1	0.000 ± 0.000	0.000 ± 0.000	0.004 ± 0.004	0.000 ± 0.000	0.001 ± 0.002	0.003 ± 0.005	0.000 ± 0.000	3.239	**0.009**
Total MUFA	0.479 ± 0.064	0.306 ± 0.081^a^	0.658 ± 0.124^ab^	0.179 ± 0.038^abc^	0.185 ± 0.043^abc^	0.582 ± 0.086^abde^	0.411 ± 0.063^bcdef^	48.109	** < 0.001**
C18:2 n-6c	0.188 ± 0.032	0.377 ± 0.088^a^	0.278 ± 0.060^ab^	0.516 ± 0.077^abc^	0.671 ± 0.121^abcd^	0.322 ± 0.077^ade^	0.411 ± 0.042^acdef^	35.158	** < 0.001**
C18:3 n-6	0.000 ± 0.000	0.001 ± 0.002	0.002 ± 0.003	0.000 ± 0.000	0.000 ± 0.000	0.000 ± 0.000	0.000 ± 0.000	2.423	**0.040**
C20:3 n-6	0.037 ± 0.019	0.037 ± 0.017	0.055 ± 0.019	0.010 ± 0.012^abc^	0.027 ± 0.018^c^	0.053 ± 0.026^de^	0.036 ± 0.018^d^	5.361	** < 0.001**
C20:4 n-6	0.433 ± 0.104	0.655 ± 0.168^a^	0.498 ± 0.196	0.128 ± 0.043^abc^	0.584 ± 0.101^d^	0.687 ± 0.189^acd^	0.686 ± 0.215^acd^	13.006	** < 0.001**
Total n-6 PUFA	0.658 ± 0.107	1.069 ± 0.234^a^	0.833 ± 0.232^b^	0.654 ± 0.101^b^	1.281 ± 0.218^acd^	1.063 ± 0.265^acde^	1.133 ± 0.253^acd^	10.637	** < 0.001**
C18:3 n-3	0.000 ± 0.000	0.000 ± 0.000	0.001 ± 0.003	0.098 ± 0.028^abc^	0.003 ± 0.006^d^	0.000 ± 0.000^d^	0.000 ± 0.000^d^	89.771	** < 0.001**
C20:5 n-3	0.000 ± 0.000	0.000 ± 0.000	0.000 ± 0.000	0.198 ± 0.042^abc^	0.011 ± 0.009^d^	0.009 ± 0.006^abcd^	0.000 ± 0.000^df^	165.265	** < 0.001**
C22:6 n-3	0.107 ± 0.029	0.158 ± 0.059	0.102 ± 0.035	0.163 ± 0.025^ac^	0.240 ± 0.077^ac^	0.244 ± 0.035^acd^	0.209 ± 0.050^ac^	12.010	** < 0.001**
Total n-3 PUFA	0.107 ± 0.029	0.158 ± 0.059	0.103 ± 0.035	0.459 ± 0.065^abc^	0.254 ± 0.083^abcd^	0.253 ± 0.038^abcd^	0.209 ± 0.050^acd^	40.425	** < 0.001**
n-6/n-3 PUFA	6.604 ± 2.113	7.447 ± 2.196	8.430 ± 1.454	1.444 ± 0.247^abc^	5.510 ± 1.784^d^	4.173 ± 0.769^ cd^	5.582 ± 1.193^ cd^	17.787	** < 0.001**
Total PUFA	0.765 ± 0.121	1.227 ± 0.281^a^	0.936 ± 0.264^b^	1.113 ± 0.143^a^	1.534 ± 0.295^abcd^	1.316 ± 0.293^ac^	1.342 ± 0.283^ac^	8.748	** < 0.001**
C18:1n9t	0.000 ± 0.000	0.000 ± 0.000	0.000 ± 0.000	0.000 ± 0.000	0.000 ± 0.000	0.000 ± 0.000	0.068 ± 0.034^abcdef^	31.490	** < 0.001**

Results expressed as mean ± SD, n = 8 per group; the significance of bold meant *P* < 0.05

^a^*P* < 0.05, compared to CON group

^b^*P* < 0.05, compared to LCSFA group

^c^*P* < 0.05, compared to MCSFA group

^d^*P* < 0.05, compared to n-3 PUFA group

^e^*P* < 0.05, compared to n-6 PUFA group

^f^*P* < 0.05, compared to MUFA group

### Changes in the markers of intestinal barrier function

**Fig. 3:**
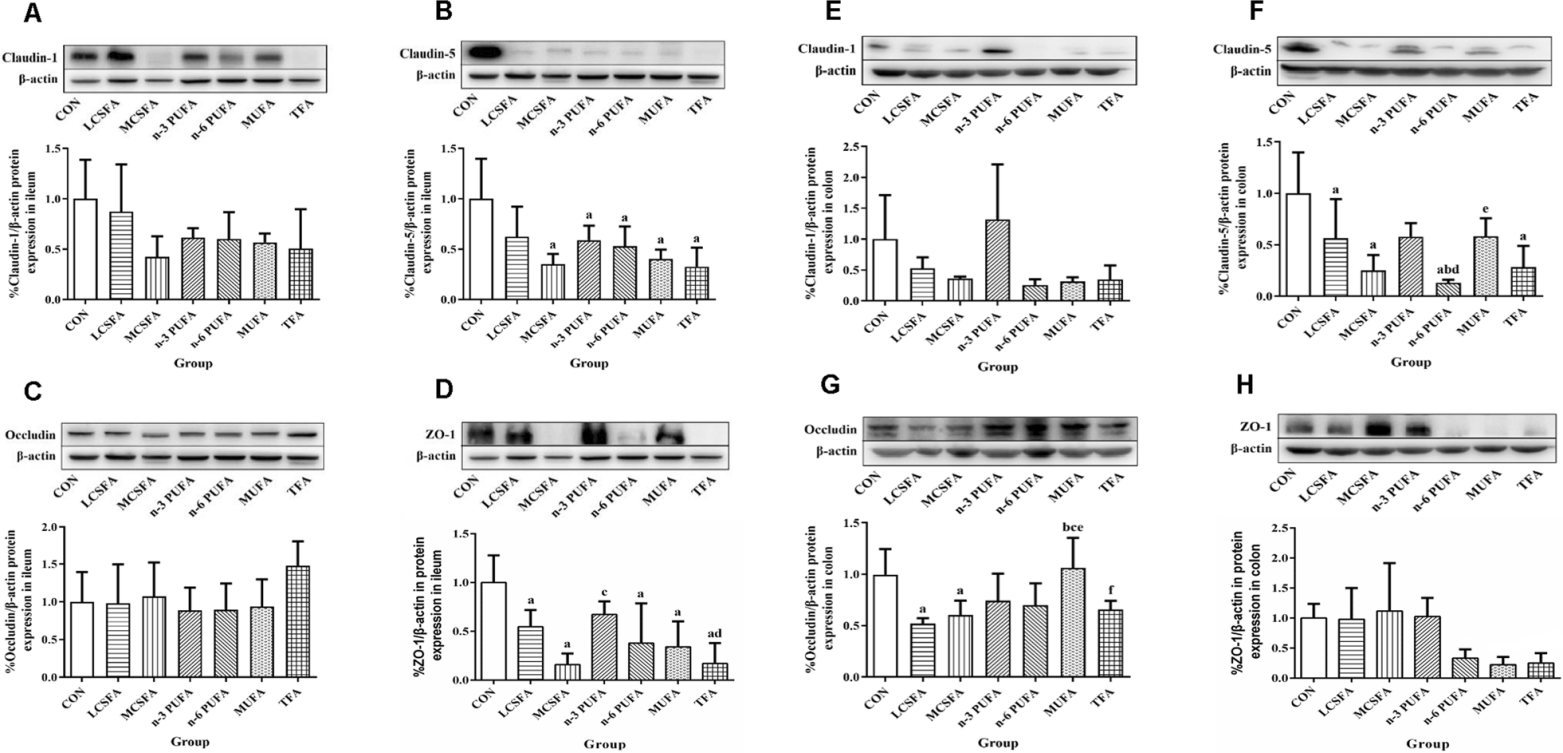
Changes in the expression of tight junction proteins in ileum and colon. **A** The expression of claudin-1 in ileum was detected by Western blotting analysis. **B** The expression of claudin-5 in ileum. **C** The expression of occuldin in ileum. **D** The expression of ZO-1 in ileum. **E** The expression of claudin-1 in colon. **F** The expression of claudin-5 in colon. **G** The expression of occuldin in colon. **H** The expression of ZO-1 in colon. n = 3, data expressed as the mean ± SD. a: *P* < 0.05, compared to CON group; b: *P* < 0.05, compared to LCSFA group; c: *P* < 0.05, compared to MCSFA group; d: *P* < 0.05, compared to n-3 PUFA group; e: *P* < 0.05, compared to n-6 PUFA group; f: *P* < 0.05, compared to MUFA group

To determine the effect of different diets on the markers of intestinal barrier function, we examined the expression of claudin-1, claudin-5, occludin, and ZO-1 proteins in the ileum and colon of mice. The Western blotting results in Fig. 3 showed that the expression level of claudin-5 in ileum decreased in the MCSFA, n-3 PUFA, n-6 PUFA, MUFA, and TFA groups, compared with that in the CON group (all *Ps* < 0.05). The claudin-5 expressions in colon in the LCSFA, MCSFA, n-6 PUFA, and TFA groups were lower than that in the CON group (all *Ps* < 0.05). The claudin-5 expression in colon in the n-6 PUFA group was lower than those in the LCSFA, n-3 PUFA, and MUFA groups (all *Ps* < 0.05). The occludin expression in colon in the CON group was higher than those in the LCSFA and MCSFA groups (all *Ps* < 0.05). Compared with that in the MUFA group, the occludin expressions in colon were decreased in the LCSFA, MCSFA, n-6 PUFA, and TFA groups (all *Ps* < 0.05). The expression level of ZO-1 in ileum in the CON group was higher than those in the LCSFA, MCSFA, n-6 PUFA, MUFA, and TFA groups (all *Ps* < 0.05). Compared with that in the n-3 PUFA group, the expression levels of ZO-1 in ileum decreased in the MCSFA and TFA groups (all *Ps* < 0.05). Based on the effect of dietary fat composition, the alteration of gut tight junction proteins in the ileum among all groups showed a higher consistency than that in the colon. The former suggested that high MCSFA and TFA diets might cause stronger damage to mucosal barrier in ileum.

### Changes in the BDNF and 5-HT of brain and intestine

**Fig. 4:**
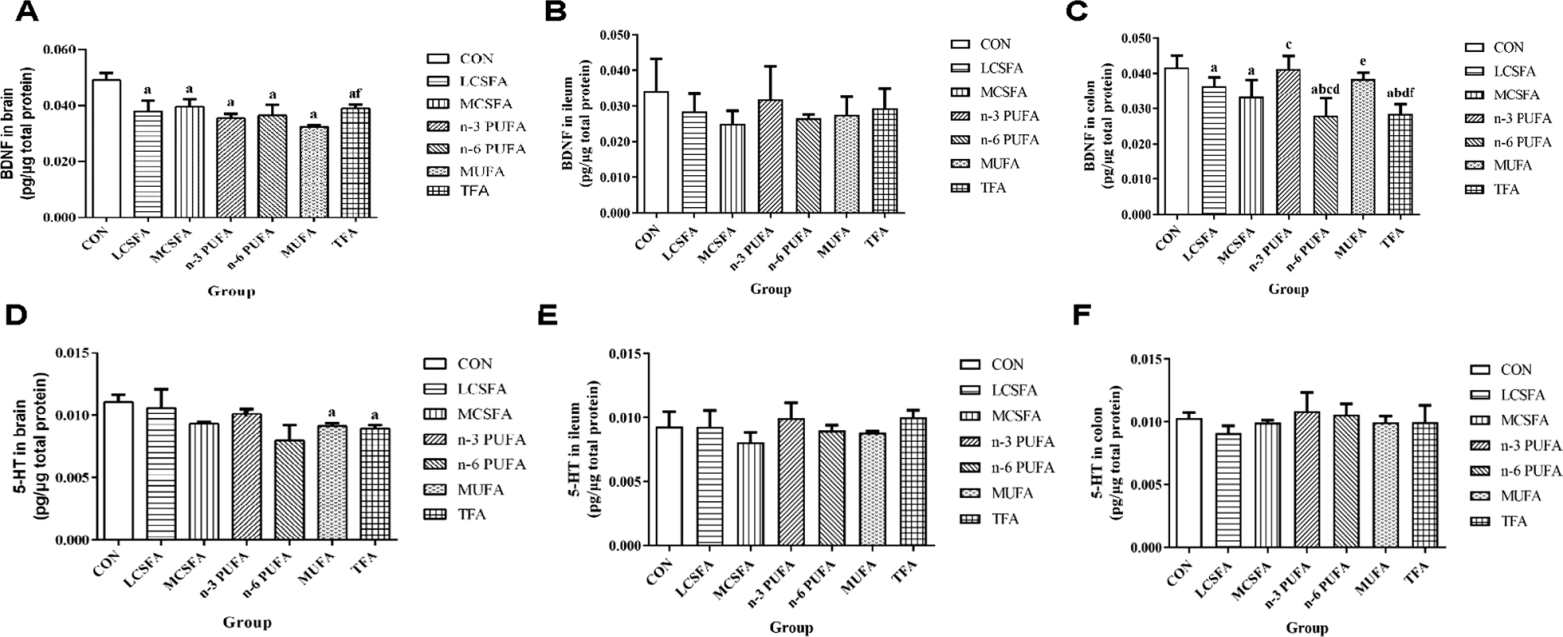
Changes in the protein levels of BDNF and 5-HT of brain, ileum, and colon. **A** The expression of BDNF in brain was detected by ELISA. **B** The expression of BDNF in ileum. **C** The expression of BDNF in colon. **D** The expression of 5-HT in brain. **E** The expression of 5-HT in ileum. **F** The expression of 5-HT in colon. n = 4, data expressed as the mean ± SD. a: *P* < 0.05, compared to CON group; b: *P* < 0.05, compared to LCSFA group; c: *P* < 0.05, compared to MCSFA group; d: *P* < 0.05, compared to n-3 PUFA group; e: *P* < 0.05, compared to n-6 PUFA group; f: *P* < 0.05, compared to MUFA group

As shown in Fig. 4, ELISA assay shows the protein expression levels of BDNF and 5-HT in brain, ileum, and colon. Compared with that in the CON group, the BDNF expression decreased in brain in other groups (all *Ps* < 0.05). The BDNF of brain in the MUFA group was lower than that in the TFA group (*P* < 0.05). Compared with that in the CON group, the BDNF of colon decreased in the LCFSA and MCSFA groups (all *Ps* < 0.05). The BDNF of colon in the n-3 PUFA group was higher than that in the MCSFA group (*P* < 0.05). The BDNF in colon in the n-6 PUFA group was lower than those in the CON, LCSFA, MCSFA, n-3 PUFA, and MUFA groups (all *Ps* < 0.05). Additionally, the BDNF of colon in the TFA group was lower than those in the CON, LCSFA, n-3 PUFA, and MUFA groups (all *Ps* < 0.05). The expression levels of 5-HT in brain decreased in the MUFA and TFA groups, compared with the CON group (all *Ps* < 0.05). Overall, the n-3 PUFA group displayed the least effect on BDNF and 5-HT in gut–brain axis.

### Changes in the leptin, insulin, and ghrelin of hippocampus and intestine

**Fig. 5:**
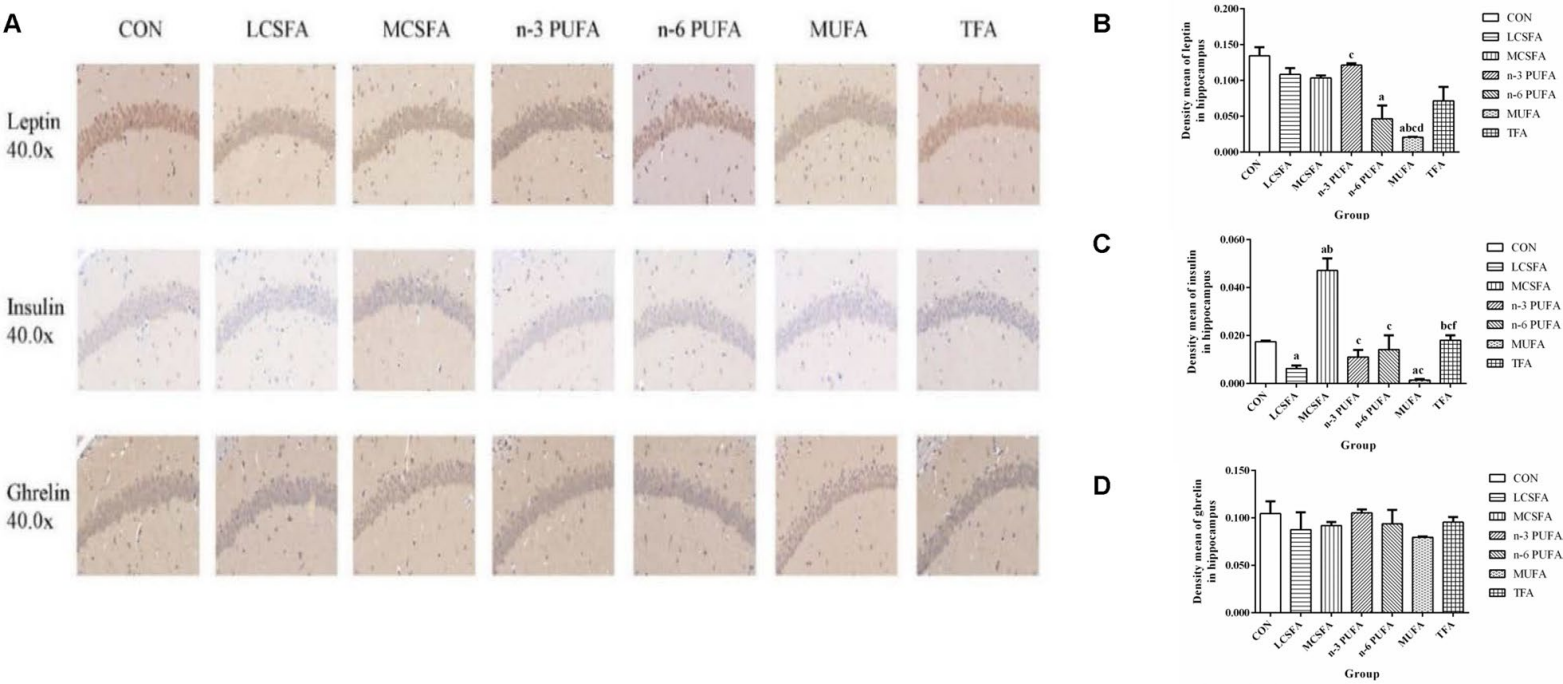
Changes in the immunoexpression of leptin, insulin, and ghrelin in hippocampus. **A** The expressions of leptin, insulin, and ghrelin in hippocampus were evaluated by immunochemistry staining. The positivity was visualized in tan. **B** The immunoexpression of leptin in hippocampus. **C** The immunoexpression of insulin in hippocampus. **D** The immunoexpression of ghrelin in hippocampus. n = 3, data expressed as the mean ± SD. a: *P* < 0.05, compared to CON group; b: *P* < 0.05, compared to LCSFA group; c: *P* < 0.05, compared to MCSFA group; d: *P* < 0.05, compared to n-3 PUFA group; f: *P* < 0.05, compared to MUFA group

**Fig. 6:**
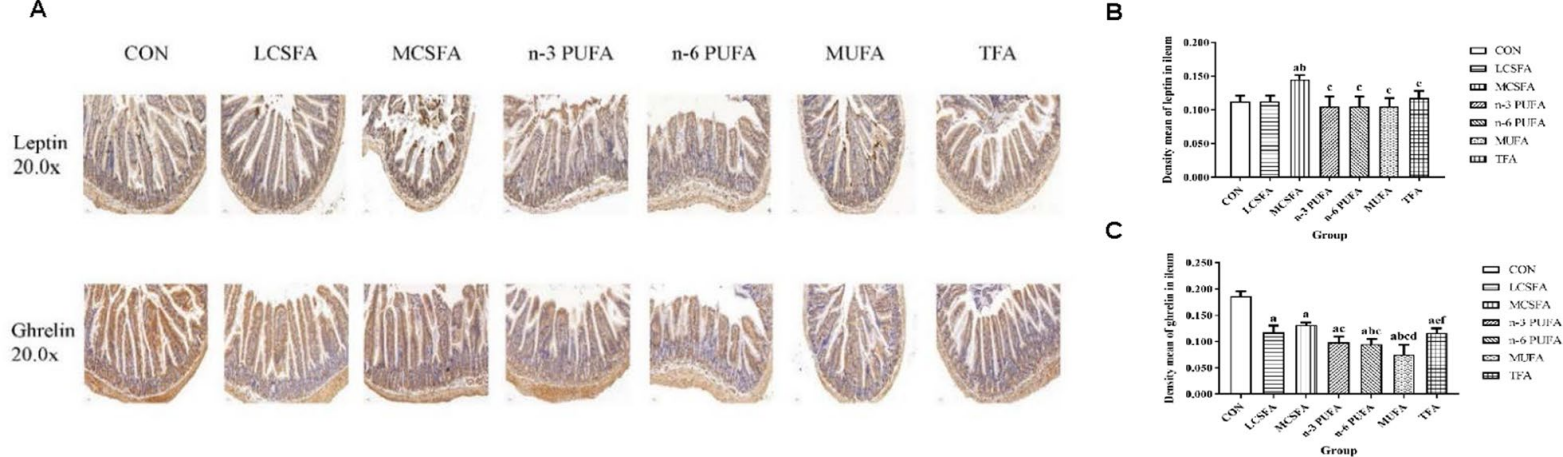
Changes in the immunoexpression of leptin and ghrelin in ileum. **A** The expressions of leptin and ghrelin in ileum were evaluated by immunochemistry staining. The positivity was visualized in tan. **B** The immunoexpression of leptin in ileum. **C** The immunoexpression of ghrelin in ileum. n = 3, data expressed as the mean ± SD. a: *P* < 0.05, compared to CON group; b: *P* < 0.05, compared to LCSFA group; c: *P* < 0.05, compared to MCSFA group; d: *P* < 0.05, compared to n-3 PUFA group; e: *P* < 0.05, compared to n-6 PUFA group; f: *P* < 0.05, compared to MUFA group

**Fig. 7:**
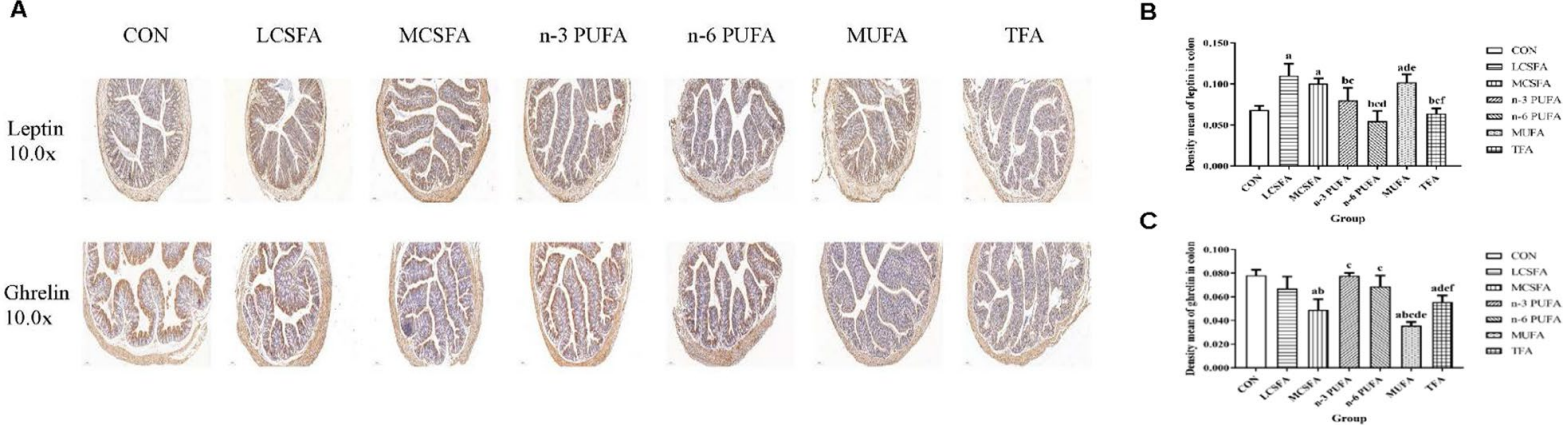
Changes in the immunoexpression of leptin and ghrelin in colon. **A** The expressions of leptin and ghrelin in colon were evaluated by immunochemistry staining. The positivity was visualized in tan. **B** The immunoexpression of leptin in colon. **C** The immunoexpression of ghrelin in colon. n = 3, data expressed as the mean ± SD. a: *P* < 0.05, compared to CON group; b: *P* < 0.05, compared to LCSFA group; c: *P* < 0.05, compared to MCSFA group; d: *P* < 0.05, compared to n-3 PUFA group; e: *P* < 0.05, compared to n-6 PUFA group; f: *P* < 0.05, compared to MUFA group

As shown in Figs. 5, 6 and 7, immunohistochemical analysis demonstrates that the leptin immunoexpression in hippocampus in the MCSFA was lower than that in the n-3 PUFA group (*P* < 0.05). The leptin of hippocampus in the n-6 PUFA group was lower than that in the CON group (*P* < 0.05). The leptin in hippocampus decreased in the MUFA group, compared with those in the CON, LCSFA, MCSFA, and n-3 PUFA groups (all *Ps* < 0.05). The insulin expressions in hippocampus decreased in the LCSFA and MUFA groups, compared with those in the CON and TFA groups (all *Ps* < 0.05). Compared with that in the MCSFA group, the insulin in hippocampus decreased in other groups (all *Ps* < 0.05). Compared with that in the MCSFA group, the leptin in ileum decreased in other groups (all *Ps* < 0.05). The ghrelin of ileum in the CON group was higher than those in other groups (all *Ps* < 0.05). The ghrelin of ileum in the n-3 PUFA group was lower than that in the MCSFA group, in contract; it was higher than that in the MUFA group (both *Ps* < 0.05). The ghrelin in ileum increased in the LCSFA, MCSFA, and TFA groups, compared with those in the n-6 PUFA and MUFA groups (all *Ps* < 0.05). The leptin expressions of colon in the CON, n-3 PUFA, n-6 PUFA, and TFA groups were lower than those in the LCSFA, MCSFA, and MUFA groups (all *Ps* < 0.05). The leptin of colon in the n-3 PUFA group was higher than that in the n-6 PUFA group (*P* < 0.05). In addition, compared with that in the MCSFA group, the levels of ghrelin of colon were increased in the CON, LCSFA, n-3 PUFA, and n-6 PUFA groups (all *Ps* < 0.05). The ghrelin of colon in the MUFA group was lower than those in other groups (all *Ps* < 0.05). The ghrelin of colon in the TFA group was lower than those in the CON, n-3 PUFA, and n-6 PUFA groups (all *Ps* < 0.05). It was obvious of above results that high MCSFA diet might upregulate the insulin expression in hippocampus and the leptin in ileum of mice.

## Discussion

The primary determinant of health is dietary components [17]. A lot of researches showed that high-fat diets may induce obesity and affect brain function [18, 19]. Our results indicated that all high-fat groups significantly increased body weights in mice, especially the n-6 PUFA group, compared with the CON group at the end of this study. Moreover, the body fat mass, such as perirenal fat, peri-testicular fat, and omental fat showed the same trend as the body weight. Massiera et al*.* indicated that a high ratio of n-6/n-3 PUFA may be a risk factor for obesity in rodents and possibly in humans [20]. Bruce-Keller et al*.* [19] demonstrated that long-term consumption of high SFA diet induced obesity in mice. Related studies showed that the consumption of TFA was related to changes in blood lipids [21] and obesity in animals and humans [22]. All these studies were similar to our outcomes. The effect of MUFA on obesity is controversial in recent researches. Some studies indicated that MUFA could reduce blood lipids in obese rats, even prevent obesity [23]. However, other studies showed that high MUFA diet improved body mass in mice [24], which was similar with our study. Compared with other high-fat diets, the high n-3 PUFA diet induced the smallest increases in the weight and body fat of mice, which is similar to these evidences showing that increased n-3 PUFA intake may prevent obesity and reduce the body fat mass of obese subjects [25–27].

High-fat diet-induced obesity leads to lipid disorders. Our results showed that the contents of C12:0, C14:0, C16:0, C22:0, total SFA, C16:1, C18:1 n-9c, total MUFA, and n-6/n-3 PUFA in the MCSFA group were markedly higher than those in most test groups. The contents of C22:6 n-3 and total n-3 PUFA were markedly lower than those in other test groups. Related researches in our subject group found that compared with healthy people, lipid disorders in the brain and blood of people with cognitive dysfunction showed high levels of SFA (especially C20:0) [28], MUFA, and n-6 PUFA [29]. In contract, lower level of n-3 PUFA (especially C22:6 n-3) was observed in those people [30], which was similar with our findings. The previous research of our group showed that the increased plasma SFA and MUFA was positively correlated with the incidence of mild cognitive impairment [28]. Reversely, in our study, the contents of C16:0, total SFA, C18:1 n-9c, total MUFA, C20:3 n-6, C20:4 n-6, total n-6 PUFA, and n-6/n-3 PUFA in the n-3 PUFA group were markedly lower than those in most test groups. Song et al*.* found that with the increased ratio of serum n-3/n-6 PUFAs, the risk of cognitive impairment in the elderly decreased [31].

The tight junction complex between endothelial cells involves transmembrane proteins (e.g. claudin-5, occludin) and scaffolding proteins (e.g. ZO-1, ZO-2), which are important for paracellular space occlusion and physical support [32]. However, high-fat diets affect the expression of these proteins to undermine the permeability and integrity of intestinal barrier. Cani et al*.* [33] demonstrated that the high-fat diet evidently increased intestinal permeability by reducing the expressions of occludin and ZO-1. Gil-Cardoso et al*.* [34] also found that the expressions of claudin-1 and ZO-1decreased in obese Wistar rats, compared with the controls. Moreover, AD related study found decreased expressions of claudin-1 and claudin-5 and increased blood–brain barrier (BBB) permeability in their 3D human neural cell culture microfluidic model [35]. However, Yuan and Willemsen et al*.* indicated that n-3 PUFA supported epithelial barrier integrity and reduced IL-4 mediated permeability [36, 37]. In our results, all high-fat diets decreased the expressions of intestinal tight junction proteins, especially high MCSFA and TFA diets, but high n-3 PUFA diet had minimal damage to the epithelial barrier, which were consistent with previous related researches.

Furthermore, our study found that the protein expressions of BDNF in brain, ileum, and colon had different degrees of reduction in all high-fat groups. BDNF is able to suppress appetite signals in the brain and prevent obesity [38]. In addition, BDNF supports synaptic plasticity and neuronal excitability, and was important for learning and memory function [39, 40]. Molteni et al*.* [41] also showed that a high-fat, refined sugar diet reduced hippocampal BDNF, neuronal plasticity, and learning ability. Wu et al*.* [12] indicated that SD rats fed long-term high-fat diet had decreased levels of BDNF in brain, which was similar to our outcome. Moreover, in our results, the levels of 5-HT in brain significantly decreased in the MUFA, and TFA groups. But the high n-3 PUFA diet had the least effect on BDNF and 5-HT in gut–brain axis. Related researches demonstrated that 5-HT could enter central nervous system through gut–brain axis, which affected brain function [42]. Our findings showed that high-fat diets might damage the gut–brain axis by modulating BDNF and 5-HT, but whether n-3 PUFA has a protective effect on this pathway needs further validation.

Leptin can reduce appetite and energy intake, and regulate central nervous system inflammation as an immunomodulatory factor [43]. Related researches indicated that high SFA diet was positively correlated with increased serum leptin in animals and humans [44], which was associated with the occurrence of AD [15]. Several studies found that leptin resistance is associated with cognitive deficits [45, 46]. It is noteworthy that our outcome of leptin in hippocampus was contrary to previous related researches, which might because high-fat diets decreased the rate of leptin transported across the BBB [47]. In our study, the insulin of hippocampus in the MCSFA group increased among all groups, and decreased in the LCSFA and MUFA groups, compared to the CON group. Perry et al*.* [48] showed that high-fat diet led to obesity and insulin resistance in rats. Some evidences indicated that insulin, like leptin, might have a key role in cognitive function through regulation of synaptic plasticity and trafficking of neurotransmitter receptors [49, 50]. The immunoexpression of ghrelin in ileum and colon presented different degrees of reduction in six test groups of our study. Zachary et al*.* [51] demonstrated that high-fat diet resulted in the permeability of BBB increasing and the active transport of ghrelin across the BBB decreasing.

Taken together, our results revealed that all high dietary fatty acid diets induced obesity accompanied by lipid disorders, intestinal barrier dysfunction, and changes in secreted cytokines from gut-brain axis including BDNF, 5-HT, leptin, insulin, and ghrelin. Among them, high MCSFA diet showed greater impact in terms of inducing abnormal changes of metabolism in gut-brain axis, and high n-3 PUFA diet had the least effect on changes in metabolism, showing that contrary to other types of high-fat diets, high MCSFA diet might be more prone to induce gut-brain axis imbalance and n-3 PUFA diet might have a protective effect on gut-brain axis.

## Data Availability

All data generated or analyzed during this study are included in this published article.

## Abbreviations

5-HT: Serotonin
AD: Alzheimer’s disease
BBB: Blood-brain barrier
BDNF: Brain-derived neurotrophic factor
CON: Control
LCSFA: Long-chain saturated fatty acid
MCSFA: Medium-chain saturated fatty acid
MUFA: Monounsaturated fatty acid
PLS: Fisher’s protected least significant difference
RIPA: Radio immunoprecipitation assay
SD: Standard deviation
TFA: Trans fatty acid
ZO-1: Zonula occludens-1
